# Prevention of cancer—Melanoma development and its diagnosis among Silesian Voivodeship residents: Preliminary results

**DOI:** 10.1097/MD.0000000000039547

**Published:** 2024-09-06

**Authors:** Józefa Dąbek, Julia Żerdka, Patryk Brasse

**Affiliations:** a Department and Clinic of Cardiology, Faculty of Health Sciences in Katowice, Medical University of Silesia in Katowice, Katowice, Poland; b Student Scientific Association at the Department and Clinic of Cardiology, Faculty of Health Sciences in Katowice, Medical University of Silesia in Katowice, Katowice, Poland.

**Keywords:** diagnosis, melanoma, prevention, risk factors

## Abstract

Melanoma is a malignant tumor with the highest growth rate in the incidence and is the leading cause of death due to skin cancers. In Poland, approximately 1500 cases of melanoma are detected annually in advanced or metastatic stages. Intensive preventive measures can contribute to its early-stage diagnosis, consequently reducing the number of fatalities. The aim of the study was to assess the occurrence of melanoma risk factors among the residents of Silesia region and their knowledge about the diagnosis and prevention of this cancer. An original questionnaire was used in the study, and its completion was anonymous. The study was conducted among the residents of the Silesian Voivodeship. A total of 400 (100%) individuals were examined. Among them were 243 women and 157 men. The participants’ ages ranged from 16 to 84 years (mean age = 34.38 ± 18.39). The participants were burdened with melanoma development risk factors such as fair skin complexion (235; 58.75%), having more than 50 pigmented lesions (158; 39.50%) and sunburns (105; 26.25%). Over 40% (166; 41.50%) of the participants had never examined their pigmented lesions. A staggering 78% (311; 77.75%) of the respondents had never undergone dermatoscopic examination, and over 50% (215; 53.75%) did not know what this examination entailed. Just under 16% (63; 15.75%) of the participants stated that their family doctor had examined their pigmented lesions, and almost % (154; 97.47%) of those with numerous pigmented lesions had never been referred to a dermatologist for dermatoscopy. The surveyed residents of the Silesian Voivodeship were burdened with numerous risk factors for melanoma development, with the most common being fair skin complexion, having more than 50 pigmented lesions, and sunburns. The knowledge of the participants regarding the diagnosis and prevention of melanoma development was insufficient, thus highlighting the necessity for conducting systematic educational initiatives in the mentioned field. These initiatives should ultimately lead to the preservation of health and life, as well as the maintenance of its high quality.

## 1. Introduction

Melanoma is a malignant tumor with the highest growth dynamics in terms of the number of cases, simultaneously being the main cause of cancer-related deaths due to skin malignancies.^[1,2]^ The incidence of melanoma is 10 to 25 new cases per 100,000 inhabitants; in the United States of America, it is 20 to 30 per 100,000; and in Australia, where a very high incidence is observed, it reaches 50 to 60 per 100,000. In recent years, there has been a dramatic increase in melanoma incidence among individuals over 60 years of age, especially in men. It is predicted that this upward trend will persist for the coming decades.^[3]^ Melanoma cases in Poland are fewer than half of the European Union average. Nevertheless, despite the lower number of cases, Poland has one of the highest mortality rates in relation to incidence in Europe and reports more deaths due to melanoma.^[4]^ Moreover, in Poland, around 1500 melanomas are detected each year at an advanced or metastatic stage. The most common phenotypic risk factor for melanoma is fair skin with a tendency to sunburn. Greater risk of developing this type of cancer also exists among individuals with a high number of pigmented lesions and large congenital and dysplastic nevi. Conversely, the most important exogenous factor is exposure to ultraviolet radiation.^[5]^ Melanoma incidence also has a genetic basis, most commonly associated with familial atypical multiple mole melanoma syndrome. In this syndrome, mutations in the cyclin-dependent kinase inhibitor 2A gene, and less frequently in cyclin-dependent kinase 4 (CDK4), are often observed. Overall, up to 45% of genetically-caused melanomas are linked to known germline mutations in the cyclin-dependent kinase inhibitor 2A or CDK4 genes.^[6,7]^ Furthermore, it has been shown that excessive exposure to ultraviolet radiation influences the genetic characteristics of melanomas. The mutation of the B-Raf protein gene in which valine (V) is substituted by glutamic acid (E) at amino acid 600 genotype is more common in patients who used tanning beds compared to those who never did, and it is also more prevalent in patients who started tanning before the age of 25 compared to those who started later in life.^[8]^ Among Poles, fair skin complexion is predominant, significantly increasing the risk of melanoma development. Intensive preventive efforts can contribute to identifying this cancer in earlier stages of its progression, consequently leading to a reduction in melanoma-related deaths. Among preventive measures aimed at early melanoma detection, clinical examinations conducted by dermatologists and enhanced with dermoscopy are particularly important due to their widespread accessibility and effectiveness. Clinical diagnosis of melanoma is based on: visual examination of the entire body’s skin, utilizing the ABCDE criteria (asymmetry, irregular border, uneven color, diameter > 6 mm, and evolution over time) to detect suspicious oncological pigmented lesions; a comparative analysis involving the search for a change that does not resemble others in the same patient and evaluating the development of pathological pigmented lesions based on available documentation. The sensitivity of clinical diagnosis performed by experienced dermatologists is estimated at around 70%.^[9]^ Expanding the clinical examination with dermoscopy increases the accuracy of assessment, achieving a sensitivity of 89% and specificity of 79% in melanoma detection.^[10]^ Dermoscopy should encompass all nevi, not just clinically suspicious ones, as it potentially allows for the detection of melanoma’s morphological features before it becomes clinically recognizable. Numerous observational studies have shown that regular whole-body screening examinations conducted by physicians are associated with a decreased frequency of advanced-stage melanomas and reduced mortality due to them.^[11–13]^ The aim of this study was to assess the prevalence of melanoma risk factors among residents of the Silesian Voivodeship and their knowledge regarding the diagnosis and prevention of this mentioned cancer.

## 2. Methods

The research commenced after obtaining approval from the Bioethics Committee of the Medical University of Silesia in Katowice for conducting the study: resolution number PCN/0022/KB1/36/21 dated May 8, 2021. The study was conducted among residents of the Silesian Voivodeship. A total of 400 (100%) individuals were surveyed. Among them were 243 women and 157 men. The participants were aged between 16 and 84 years (mean = 34.38 ± 18.39). For the study, a custom-designed questionnaire was utilized, and the completion of the questionnaire was anonymous and voluntary. General information was collected (age, gender, place of residence, and employment), along with the presence of risk factors for melanoma and its prevention among the participants. The questionnaire also included inquiries about the involvement of primary care physicians in diagnosing and undertaking preventive measures against the mentioned cancer. Paper-based questionnaires were distributed in workplaces, primary schools, higher education institutions, and retirement homes (with the consent of facility managers and participants) located within the Silesian Voivodeship. Completed surveys were stored collectively, shuffled in a dedicated folder within a locked drawer. They were only removed from this location when entering data into a spreadsheet. As a result, the identification of respondents was not possible, ensuring complete anonymity for the participants. All methods applied in the study were carried out in accordance with appropriate guidelines and regulations. Data were collected and processed using Microsoft Excel. The analyzed results were presented in numerical and percentage values. The results presented in tables were divided based on gender and individual risk factors for melanoma that were present in the study group. To calculate statistical significance, the chi-square test was used. The null hypothesis negated the existence of a relationship between variables. The critical level of significance was set at *P* < .05.

## 3. Results

### 3.1. General characteristics of the study group

The overall characteristics of the study group, including gender, age, place of residence, and education, are presented in Table 1.

**Table 1:** General characteristics of the study group.

Study group n = 400 (100%)			
Variable		n	%
Sex	Female	243	60.75
	Male	157	39.25
Age	24 or less	187	46.75
	25–49	137	34.25
	50–65	40	10
	66 or more	36	9
Place of residence	Countryside	208	52
	City up to 100,000 inhabitants	117	29.25
	City above 100,000 inhabitants	75	18.75
Education	Elementary	104	26.00
	Vacational	15	3.75
	Secondary	144	36
	Higher	137	34.25

n: sample size.

The majority of the study group consisted of women and individuals under 50 years of age, with rural areas being the predominant place of residence. Those with higher and secondary education accounted for over 70% of all respondents.

### 3.2. Risk factors for melanoma development in the study group

The characteristics of the study group, taking into account the presence of risk factors for melanoma development, are presented in Table 2.

**Table 2:** Presence of risk factors for melanoma development (multiple choices possible).

Study group		
n = 400 (100%)		
Risk factors for melanoma	n	%
Fair skin complexion	235	58.75
Presence of more than 50 pigmented lesions	158	39.50
Light eye color	127	31.75
Sunburns of the skin	105	26.25
Light hair color	86	21.50
Sunburns during childhood	79	19.75
Excessive exposure to sunlight	77	19.25
Family history of skin melanoma	61	15.25
Numerous freckles	54	13.5
Age above 65	36	9.00
Use of tanning beds	31	7.75
Immunosuppression	19	4.75
History of previous skin melanoma	16	4.00

n: sample size.

The participants were affected by risk factors for melanoma development, such as: fair skin complexion (235; 58.75%), having more than 50 pigmented lesions (158; 39.50%), sunburns (105; 26.25%), family history of melanoma (58; 14.50%), and tanning bed usage (31; 7.75%). Additionally, 16 (4%) respondents had a history of melanoma.

### 3.3. Self-examination of pigmented lesions by the participants

The characteristics of the study group, including the method and frequency of self-examination of pigmented lesions, are presented in Table 3.

**Table 3:** Method and frequency of self-monitoring of pigmented lesions (multiple choices possible in part 1).

Study group n = 400 (100%)				
Variable	Males (n = 157; 39.35%)		Females (n = 243; 60.75%)	
	n	%	n	%
Sample size (n, % of study group)	157	100%	243	100%
1. How do you control your pigmented lesions?				
Regularly, I control my lesions	57	36.31	129	53.09
A friend or family member examines my lesions	25	15.92	45	18.52
I photograph my lesions	5	3.18	16	6.58
The doctor looks at my lesions	17	10.83	46	18.93
I don’t control my lesions	82	52.23	84	34.57
2. How often do you control your pigmented lesions?				
Once a month	22	14.01	44	18.11
Once every 6 months	17	10.83	57	23.46
Once a year	18	11.46	28	11.52
Less than once a year	18	11.46	30	12.35
Never	82	52.23	84	34.57

n: sample size.

Regular self-examination of pigmented lesions was reported by 53% (129; 53.09%) of women and 36% (57; 36.30%) of men. A statistically significant correlation was found between performing self-skin examinations and gender (*P* = .024). Only 46 (18.93%) women and 17 (10.83%) men stated that a doctor had examined their skin for the presence of pathological lesions. Over 40% (166; 41.50%) of participants had never examined their pigmented lesions. Women performed self-skin examinations more often than men. Self-examination of the skin at least once a year was done by over 50% (129; 53.09%) of women and 36% (57; 36.31%) of men, while 44 (18.11%) women and 22 (14.01%) men did this once a month.

### 3.4. The frequency of performing preventive dermatoscopic examinations by the respondents

**Figure 1.**
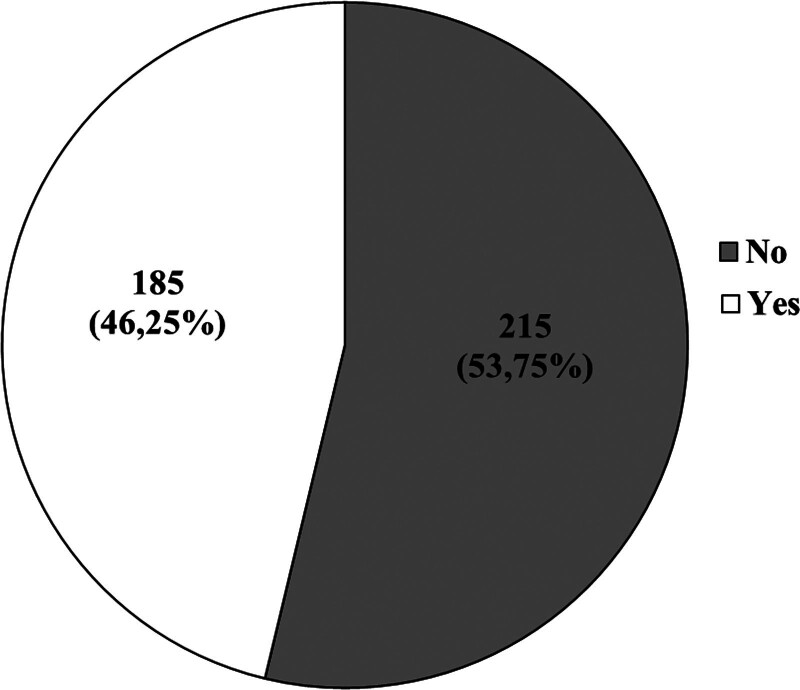
Do you know what a dermatoscopic examination is?

**Figure 2.**
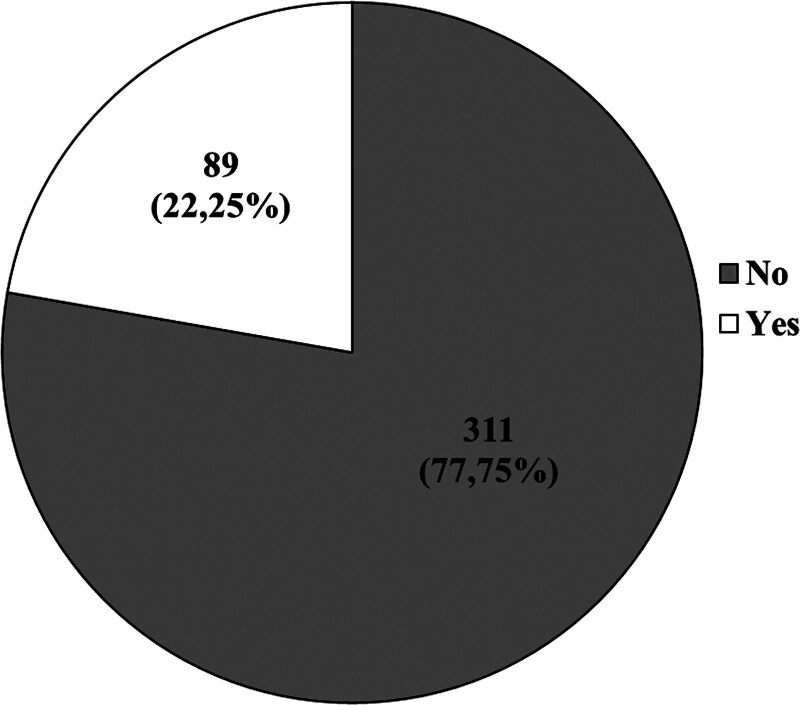
Have you ever had a dermatoscopic examination?

Figures 1 and 2 present the respondents’ answers to the questions of whether they know what a dermatoscopic examination is and if they have ever had 1. Table 4, on the other hand, provides the characteristics of the study group, considering the frequency of undergoing dermatoscopic examinations.

**Table 4:** Frequency of dermatoscopic examination by respondents.

Study group n = 400 (100%)		
Frequency of dermatoscopic examination	n	%
I get tested more than once a year	3	0.75
I get tested once a year	11	2.75
I get tested every 2–3 years	9	2.25
I get tested every 4–5 years	6	1.50
I’ve only had them once in my life	45	11.25
I test myself less than every 5 years	15	3.75

n: sample size.

As much as 80% (311; 77.75%) of respondents had never undergone a dermatoscopic examination, and over 50% (215; 53.75%) did not know what it entails. Less than 3% (11; 2.75%) of participants underwent dermatoscopic examinations once a year, and 11% (45; 11.25%) had it done once in their lifetime.

### 3.5. The frequency of performing self-examination of pigmented lesions and dermatoscopic examinations based on the risk factors for melanoma development

Table 5 presents the self-declared frequency of performing self-examination of pigmented lesions and dermatoscopic examinations, based on the risk factors for melanoma development among the respondents.

**Table 5:** Frequency of performing skin self-examination based on the risk factors for melanoma development.

Study group								
n = 400 (100%)								
Variable	Risk factors for the development of melanoma							
	People with melanoma (n = 16; 0.04%)		People with a family history of skin melanoma (n = 61; 15.25%)		People with pigment lesions in a quantity above 50 (n = 158; 39.50%)		People with fair skin (n = 235; 58.75%)	
	n	%	n	%	n	%	n	%
Sample size (n; % of study group)	16	100	61	100	158	100	235	100
1. How often do you control your pigmented lesions								
Once a month	0	0	3	4.92	19	12	35	15
Once every 6 months	5	31.3	16	26.23	29	18	41	17
Once a year	3	18.8	5	8.2	21	13	29	12
Less	3	18.8	12	19.67	25	16	26	11
2. How often you perform a dermatoscopic examination								
I get tested more than once a year	0	0	0	0	0	0	0	0
I get tested once a year	0	0	0	0	6	3.8	8	3
I get tested every 2–3 years	2	12.5	2	3.28	5	3.2	3	1
I get tested every 4–5 years	1	6.25	0	0	0	0	0	0
I’ve only had them once	2	12.5	1	1.64	5	3.2	6	3
I examine less often	2	12.5	2	3.28	6	3.8	7	3

n: sample size.

Only 55% (131; 55.74%) of individuals with fair skin complexion performed self-skin examinations, and as many as 30% (5; 31.25%) of respondents with a personal history of melanoma had never checked their pigmented lesions. Dermatoscopic examinations done once a year were performed by 4% (6; 3.8%) of respondents with numerous pigmented lesions, 3% (8; 3.4%) with fair skin complexion, and 0% of respondents with a familial or personal history of melanoma.

### 3.6. Reasons that influenced or deterred the respondents from undergoing follow-up dermatoscopy examinations by a dermatologist

**Figure 3.**
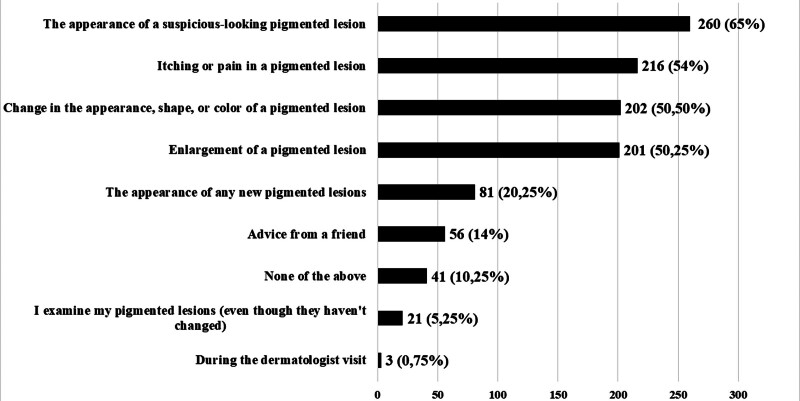
Motivations of the respondents for a dermatoscopic examination (multiple choices possible).

Table 6 presents the reasons why the respondents never had their pigmented lesions checked by a dermatologist using dermatoscopy. Table 7 displays the reasons why they did it less frequently than once a year. Figure 3 shows what, according to the respondents, would motivate them to visit a dermatologist for a follow-up dermatoscopic examination.

**Table 6:** Reasons for not performing a dermoscopy examination (multiple choices possible).

Study group n = 400 (100%)		
Why have you never had your pigmented nevi checked by a dermatologist using a dermatoscope?	n	%
I believe that my pigmented lesions do not arouse suspicion	175	43.75
I did not know that pigmented lesions should be controlled by a dermatologist by dermatoscopic examination	154	38.5
I do not belong to the risk group	45	11.25
I don’t have time for that	39	9.75
I don’t want to control my lesions	34	8.5
I can’t afford such a test	22	5.5
I’m afraid to control my change	20	5
I have no pigmented lesions	15	3.75
I don’t have the possibility to get to this examination	6	1.5

n: sample size.

**Table 7:** Reasons why the respondents had a dermatoscopic examination less than yearly (multiple choices possible).

Study group n = 400 (100%)		
Why do you check your pigmented nevi less than once a year?	n	%
I did not know that the dermatoscopic examination should be repeated once a year	50	12.5
I believe that my pigmented lesions have not changed since the last examination	44	11
Too long waiting time for an examination	17	4.25
I don’t have time for that	15	3.75
Too high cost of private dermatoscopic examination	14	3.5
I do not want to control pigmented lesions so often	9	2.25
I’m afraid to control my change	8	2

n: sample size.

As a reason for not undergoing follow-up dermatoscopic examinations of skin lesions, almost 40% (154; 38.5%) of respondents cited a lack of knowledge that pigmented lesions should be examined by a dermatologist using this specific method. Over 40% (175; 43.75%) stated that their pigmented lesions did not raise any suspicions, and 5% (20; 5%) were afraid to undergo the examination. More than 12% (50; 12.5%) of respondents were unaware that dermatoscopic examinations should be done once a year. As reasons for less frequent examinations, over 4% mentioned the long waiting time for a dermatologist appointment, and 11% (44; 11%) mentioned the absence of changes in their lesions since the last examination. Over 50% of respondents would be motivated to visit a dermatologist for a follow-up examination due to factors such as enlargement, changes in appearance, itching, and pain in pigmented lesions.

### 3.7. Actions related to melanoma prevention taken by family doctors in the examined community

**Figure 4.**
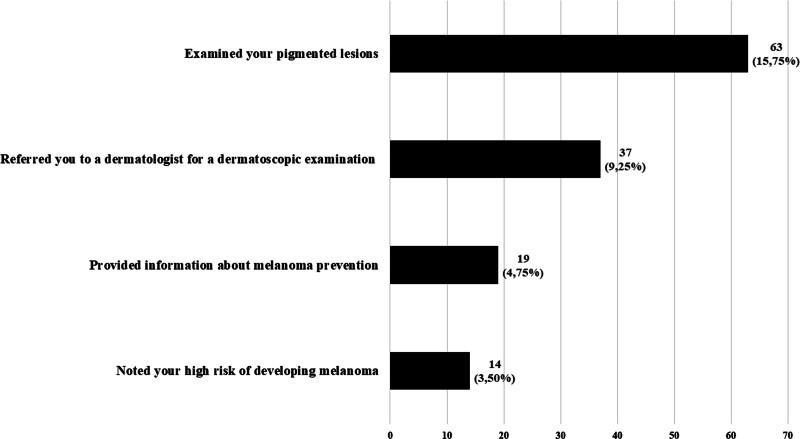
Actions of family doctors in the scope of melanoma prevention (multiple choice possible).

Figure 4 depicts the actions related to melanoma prevention undertaken by family doctors in the examined community, while detailed data regarding knowledge of the most common risk factors are presented in Table 8.

**Table 8:** Family doctors’ role in preventing melanoma development in at-risk individuals (multiple choice possibility).

Study group						
n = 400 (100%)						
Variable	Risk factors for the development of melanoma					
	Fair complexion (n = 235; 58.75%)		Numerous pigmented lesions		Melanoma of a family member’s skin (n = 61; 15.25%)	
			in the number of more than 50 (n = 158; 39.50%)			
	n	%	n	%	n	%
Sample size (n; % of study group)	235	100%	158	100%	61	100%
Action taken by the doctor						
Examined your pigmented lesions	16	6.81	13	8.23	4	6.56
Provided information on melanoma prevention	3	1.28	0	0	1	1.64
Drew attention to your high risk of melanoma	2	0.85	2	1.27	0	0
Referred you to a dermatologist for a dermatoscopic examination	3	1.28	4	2.53	2	3.28

n: sample size.

Over 97% (154; 97.47%) of the examined individuals with numerous pigmentary lesions have never been referred to a dermatologist for dermatoscopy. Only 1 person, among those with a close family member with a history of skin melanoma, received information from a doctor regarding its prevention.

## 4. Discussion

This study was conducted with the aim of documenting melanoma risk factors among the surveyed residents of the Silesian Voivodeship and assessing the preventive measures taken against this malignant tumor. Unfortunately, it revealed that melanoma prevention is not widely practiced, underscoring the need for interventions aimed at improving behaviors that prevent the development of this particular cancer, especially among patients in higher-risk groups. This appears to be particularly important considering the rising incidence of melanoma and high treatment costs. In addition to implementing targeted educational interventions, greater emphasis should be placed on enhancing the quality of guidance provided by physicians and introducing appropriate guidelines for comprehensive skin examinations.

This study was conducted to document melanoma risk factors among the surveyed residents of the Silesian Voivodeship and evaluate the preventive measures taken against this malignant tumor. Unfortunately, it revealed that melanoma prevention is not widely practiced, highlighting the need for interventions aimed at improving behaviors that prevent the development of this cancer, especially among patients in higher-risk groups. This seems particularly important given the rising incidence of melanoma and its high treatment costs. Despite numerous risk factors, the surveyed population of Silesian residents seldom engaged in skin self-examinations or regular dermatoscopic checkups. Only 55% (131; 55.74%) of individuals with fair skin complexion performed skin self-examinations, and as much as 30% (5; 31.25%) with a history of melanoma had never checked their pigmented lesions. Annual dermatoscopic examinations were carried out by 4% (6; 3.8%) of respondents with numerous pigmented lesions, 3% (8; 3.4%) with fair skin complexion, and 0% of participants with a familial or personal history of melanoma.

Studies conducted in California on melanoma risk factors showed that 31.5% of respondents had fair skin complexion, and 2.4% had more than 10 pigmented nevi. Those with fairer skin and sunburns more frequently reported recent skin examinations, and respondents with 10 or more pigmented nevi were almost 2.5 times more likely to have undergone prior melanoma screening than those without any nevi. Respondents with a personal history of melanoma were 7 times more likely to have sought screening. The history of melanoma in immediate family members and spouses was also significantly associated with recent screening.^[14]^ Other studies evaluating the frequency of skin self-examination indicated that 23% to 61% of the surveyed population reported performing self-examinations at least once a year, with higher rates in Australia and the United States, and among those with a familial or personal history of melanoma. Unfortunately, the same studies revealed that nearly 18% of respondents who had melanoma would disregard new or changing pigmented lesions and not seek medical attention.^[15]^ Analyses conducted in Cyprus and the USA also indicated that the presence of melanoma risk factors in respondents correlated with more frequent skin self-examinations.^[16,17]^ Annual clinical skin examinations by dermatologists were also more common among individuals with a familial (15% of men and 14% of women) and personal history (83% of men and 86% of women) of melanoma.^[18]^ These findings suggest that melanoma prevention among the surveyed residents of the Silesian Voivodeship, who are burdened by numerous risk factors, is suboptimal and lower than in other studied populations. It is possible that for some reason, knowledge about the risks of this cancer and its prevention did not reach the surveyed population or was disregarded. Therefore, further investigation of this issue is necessary to improve melanoma prevention efforts among the population of the Silesian Voivodeship.

In our own study, regular self-examination of pigmented lesions was reported by 32% (129; 53.09%) of women and 13% (57; 36.31%) of men. More than 40% (166; 41.50%) of participants had never examined their pigmented lesions. Women performed skin self-examination more frequently than men. More than 50% (129; 53.09%) of women and 36% (57; 36.31%) of men conducted self-examination at least once a year, while 44 (18.11%) women and 22 (14.01%) men did it monthly.

In a study conducted among Cypriots, 59% of respondents performed skin self-examination, of which 21% did it monthly and 7% did it annually.^[16]^ A study in Romania revealed that half of the participants conducted skin self-examinations, with one-third of them doing it regularly (22.48% monthly and 13.28% annually).^[15]^ The data from our own study indicate that women performed skin self-examination more frequently than men. Similar results were obtained by Kasparian et al in their research on respondents without a history of melanoma: skin self-examination was more frequently reported by women (70%) than men (67%), and one-third of both women and men without a history of melanoma had never examined their pigmented lesions.^[18]^ However, in the United States, men (16%) performed skin self-examination more often than women (13%).^[17]^ Twin studies also indicated that 29% of women and 36% of men had ever conducted skin self-examinations. Female gender was associated with a lower likelihood of recent skin screening.^[14,17]^

Based on the aforementioned data, social campaigns to educate about skin self-examination are needed in many places worldwide, as this practice is still not widespread enough. In Poland, especially in the Silesian Voivodeship, it is important for knowledge about melanoma prevention to be widely disseminated among the entire population and reach men. For comparison, a study conducted among dermatologists, a group with very high knowledge about melanoma and its prevention, showed that 72% of respondents routinely examined their pigmented lesions, with 25.4% performing self-examination monthly, 24.5% every 6 months, and 17.2% annually. Among all respondents from that study, 67% conducted skin examinations at least once a year, and this was not significantly related to gender.^[19]^ Routine skin self-examination is crucial, as it is associated with significantly reduced mortality due to melanoma through early detection. About 75% of melanomas are detected by patients themselves or by their spouses, friends, and others not related to medicine. Dermatology and oncology societies recommend monthly comprehensive skin self-examination in high-risk groups such as individuals with a personal history of melanoma or with atypical mole syndrome. Despite such recommendations, the rate of systematic self-examination of the skin among patients is low, which could be improved through proper education.^[20]^

Based on our own study, it was found that a significant 80% (311; 77.75%) of participants had never undergone dermoscopy, and over 50% (215; 53.75%) were unaware of what the mentioned examination entails. Less than 3% (11; 2.75%) of respondents had dermoscopy once a year, while 11% (45; 11.25%) had it once in their lifetime. Among individuals with a family history of melanoma, only 43.75% (7; 43.75%) had undergone dermoscopy.

In another study, it was shown that a larger proportion, 25.4% of participants, had undergone dermoscopy at least once in their lifetime, and nearly 40% had sought advice from a dermatologist or another specialist to assess their pigmented lesions. Furthermore, one-tenth of respondents lacked knowledge about dermoscopy, in comparison to half of the respondents from our own study.^[21]^

In a study conducted among individuals with a family history of melanoma, it was demonstrated that 43% of respondents had clinical skin examinations at least once a year, and 17% had them every 2 years. Meanwhile, 27% of participants had never had their skin examined by a doctor.^[22]^ In comparison to the mentioned studies, the analyzed group of Silesian residents exhibited significantly lower adherence to preventive skin examinations, which is particularly concerning among individuals with a family history of melanoma, as they belong to a high-risk group for the development of this cancer.

As reasons for not undergoing routine dermatoscopic examinations, nearly 40% (154; 38.5%) of respondents cited a lack of knowledge that pigmented lesions should be examined by a dermatologist using this method. Another 40% (175; 43.75%) stated that their pigmented lesions did not raise suspicion, and 5% (20; 5%) were afraid to have them examined. More than 12% (50; 12.5%) of participants were unaware that dermoscopy should be performed annually, and as a reason for less frequent examination, over 4% mentioned the long waiting time for a dermatologist’s appointment, and 11% (44; 11%) cited the absence of changes in their pigmented lesions since the last examination.

For over 50% of respondents, enlarging, changing appearance, itching, pain in a current pigmented lesion, or the appearance of a new suspicious-looking mole would motivate them to seek a follow-up dermatologist’s visit. A study from Romania indicated that the appearance of a new or changing pigmented lesion would prompt 29% of respondents to visit a dermatologist and 26% to visit a family doctor, yet 35% of participants would ignore these symptoms.^[15]^

In another study, 52% of respondents believed that bleeding from a pigmented lesion was the only reason to consult a doctor.^[23]^ These are concerning findings, as bleeding is not a common symptom and might not even occur, leading to delayed or missed skin cancer diagnoses.

Studies conducted among medical students, a group with above-average knowledge of melanoma, revealed that their motivations for skin examinations included early detection of skin cancer (54%), fear of cancer (31%), cancer in relatives or friends (11%), and doctor’s recommendations (22%). As reasons for not undergoing skin examinations, 3% of students mentioned fear of a diagnosis.^[24]^

Among other reasons for not undergoing clinical dermatological examinations, factors included lack of time, forgetfulness, belief that it is not important, and the belief that they didn’t have suspicious pigmented lesions.^[8]^ In our own study, over 40% of respondents also mentioned that their pigmented lesions did not raise suspicion.

This raises the question of whether patients are adequately qualified to assess the oncological suspicion of pigmented lesions. Although skin self-examination is simple and effective in detecting melanoma, examinations involving doctors are crucial for early melanoma detection, leading to better outcomes for this cancer.^[11]^

In studies conducted among the rural poor population in Wielkopolska concerning preventive examinations, dermatoscopy was the least frequently performed examination (7.4%). Barriers to participating in preventive examinations included lack of time, lack of need or a sense of health (32.8%), long waiting times (17.2%), and fear of costs (9%).^[25]^ In our own study, high examination costs were a problem for 5.5% of the surveyed group, nearly 10% of respondents cited a lack of time, and 4.25% mentioned the excessively long waiting time for an appointment. Therefore, proper promotion of preventive skin examinations is crucial through education and informative campaigns, free screening for high-risk individuals, referrals for dermoscopy by family doctors, official guidelines disseminated by healthcare professionals, mobile screening units in areas with limited healthcare access offering free skin examinations and education about melanoma and other skin cancers. This is especially important considering that almost 40% of respondents were unaware that pigmented lesions should be examined by a dermatologist using dermoscopy. This constitutes a significant portion of the surveyed group, although nearly 77% of Turkish students were unaware of skin cancer screening examinations.^[26]^

Interestingly, 5% of respondents were afraid of dermoscopy, and in another study, 7% of individuals reported it as unpleasant.^[27]^ Dermoscopy is a painless procedure, and this should be emphasized in conversations with patients. While a comprehensive dermoscopic examination includes checking areas such as the oral cavity, eye conjunctiva, anus, and genitalia, which could be embarrassing for the patient, many other routine medical examinations are far less comfortable. Hence, patient communication and explaining the essence of the examination are crucial to maintain the accuracy of dermatological skin examinations.

Over 97% (154; 97.47%) of participants with numerous pigmented lesions had never been referred to a dermatologist for dermoscopy. Only 46 (18.11%) women and 17 (10.83%) men reported that a doctor had examined their skin for pathological lesions. Among those with a close family member with a history of melanoma, only 1 person received information about melanoma prevention from a doctor.

Studies conducted in Romania also revealed infrequent clinical skin examinations performed by doctors. Within a year, only 13.8% of participants in the control group and 26.4% with their own history of melanoma had undergone examinations.^[15]^ In contrast, in Australia, skin examinations conducted by doctors were more prevalent, involving around one-third of the study group within the last 12 months. Skin examinations were more common among individuals with greater sensitivity to ultra violet radiation, lighter skin tones, higher awareness of skin cancer risk, and among females.^[28]^

In a study of American military veterans, a group at increased risk of melanoma due to exposure to ultra violet radiation and barriers to sun protection during military actions, where high rates of sunburns and overrepresentation of older White men were present, less than one-third (30.88%) reported ever having a skin examination by a doctor. Among respondents reporting skin examination by a doctor, 35% had a family history of skin cancer, 39% had moderate to severe sunburns, and 30% used tanning beds.^[29]^ In comparison to the mentioned analyses, individuals with risk factors for melanoma in the surveyed group of Silesian residents were provided insufficient healthcare and received rare preventive actions.

In studies among patients with a history of squamous cell skin cancer, doctors were the main source of information for 24.4% of patients, and doctor’s recommendations increased the likelihood of taking preventive actions. In total, 38.5% of respondents reported skin assessments performed by doctors before cancer diagnosis (95.2% by dermatologists and 2.4% by general practitioners). Overall, 76.7% of patients declared receiving medical advice, with the most frequent suggestions being regular skin examinations by doctors (55.1%). Only 3.3% of patients mentioned that a doctor recommended self-examination of the skin.^[30]^

In our study, only 1 person with a close family member with a history of melanoma received information about melanoma prevention from a doctor. In another study where 41% of respondents lost a family member due to melanoma, the majority reported discussing the family history of melanoma with a doctor. Half of the respondents noted receiving recommendations from healthcare professionals to undergo skin examinations by a doctor, and 59% received recommendations for self-examination of pigmented lesions. However, only 34% emphasized that the clinician instructed them on how to properly perform self-examinations.^[22]^

Screening individuals with a family history of melanoma is crucial, as about 7 to 15% of melanoma patients have a family history in this direction, and the risk of developing melanoma doubles if a first-degree relative has previously been diagnosed with melanoma. Most of the risk is likely related to family members’ sun exposure, and less so to inheritance.^[6]^ Proper training of general practitioners in examining pigmented lesions would improve melanoma prevention in Poland, as they are in the most common contact with patients. It is crucial that additional training for general practitioners in skin examination is free and widely accessible. Patients with melanoma risk factors should also be included in regular skin cancer prevention programs led by dermatologists, as they possess the greatest knowledge and skills in melanoma recognition among all medical specialists.

## 5. Conclusions

1. The residents of the Silesian Voivodeship were exposed to numerous risk factors for melanoma development, of which the most common were: fair complexion, having more than 50 pigmented lesions, and sunburn.
2. The knowledge of the surveyed individuals regarding the diagnosis and prevention of melanoma development was inadequate. As a result, there is a necessity to carry out systematic educational activities in the mentioned area, ultimately leading to the preservation of health and life, as well as maintaining its good quality.

## Author contributions

**Conceptualization:** Józefa Dąbek, Julia Żerdka, Patryk Brasse.

**Data curation:** Józefa Dąbek, Julia Żerdka, Patryk Brasse.

**Formal analysis:** Józefa Dąbek, Julia Żerdka, Patryk Brasse.

**Investigation:** Julia Żerdka, Patryk Brasse.

**Resources:** Julia Żerdka, Patryk Brasse.

**Methodology:** Józefa Dąbek, Julia Żerdka, Patryk Brasse.

**Software:** Julia Żerdka, Patryk Brasse.

**Supervision:** Józefa Dąbek.

**Validation:** Józefa Dąbek.

**Visualization:** Julia Żerdka, Patryk Brasse.

**Writing – original draft:** Julia Żerdka, Patryk Brasse.

**Writing – review & editing:** Józefa Dąbek.

## References

[1] Giusti F Martos C Adriani S. The Joint Research Centre-European Network of Cancer Registries Quality Check Software (JRC-ENCR QCS). Front Oncol. 2023;13:1250195. 37965471 10.3389/fonc.2023.1250195 PMC10641391

[2] Arnold M Singh D Laversanne M. Global burden of cutaneous melanoma in 2020 and Projections to 2040. JAMA Dermatol. 2022;158:495–503. 35353115 10.1001/jamadermatol.2022.0160 PMC8968696

[3] Whiteman DC Green AC Olsen CM. The Growing burden of invasive melanoma: projections of incidence rates and numbers of new cases in six susceptible populations through 2031. J Invest Dermatol. 2016;136:1161–71. 26902923 10.1016/j.jid.2016.01.035

[4] Ferlay J Soerjomataram I Ervik M. GLOBOCAN 2012 v1.0, Cancer Incidence and Mortality Worldwide. International Agency for Research on Cancer. 2013. Available at: http://globocan.iarc.fr. Access date June, 20 2023.

[5] Garbe C Amaral T Peris K. European consensus-based interdisciplinary guideline for melanoma. Part 1: diagnostics: Update 2022. Eur J Cancer. 2022;170:236–55. 35570085 10.1016/j.ejca.2022.03.008

[6] Djavid AR Stonesifer C Fullerton BT. Etiologies of melanoma development and prevention measures: a review of the current evidence. Cancers (Basel). 2021;13:4914. 34638397 10.3390/cancers13194914 PMC8508267

[7] Eckerle Mize D Bishop M Resse E Sluzevich J. Familial Atypical Multiple Mole Melanoma Syndrome. In: Cancer Syndromes. Bethesda (MD): National Center for Biotechnology Information (US); 2009. 21249757

[8] Burbidge TE Bastian BC Guo D. Association of indoor tanning exposure with age at melanoma diagnosis and BRAF V600E Mutations. J Natl Cancer Inst. 2019;111:1228–31. 30923800 10.1093/jnci/djz048 PMC6855935

[9] Gachon J Beaulieu P Sei JF. First prospective study of the recognition process of melanoma in dermatological practice. Arch Dermatol. 2005;141:434–8. 15837860 10.1001/archderm.141.4.434

[10] Kittler H Pehamberger H Wolff K Binder M. Diagnostic accuracy of dermoscopy. Lancet Oncol. 2002;3:159–65. 11902502 10.1016/s1470-2045(02)00679-4

[11] Kovalyshyn I Dusza SW Siamas K Halpern AC Argenziano G Marghoob AA. The impact of physician screening on melanoma detection. Arch Dermatol. 2011;147:1269–75. 21768448 10.1001/archdermatol.2011.181

[12] Pollitt RA Geller AC Brooks DR Johnson TM Park ER Swetter SM. Efficacy of skin self-examination practices for early melanoma detection. Cancer Epidemiol Biomarkers Prev. 2009;18:3018–23. 19861521 10.1158/1055-9965.EPI-09-0310

[13] Katalinic A Waldmann A Weinstock MA. Does skin cancer screening save lives?: an observational study comparing trends in melanoma mortality in regions with and without screening. Cancer. 2012;118:5395–402. 22517033 10.1002/cncr.27566

[14] Miller KA Langholz BM Zadnick J. Prevalence and predictors of recent skin examination in a population-based twin cohort. Cancer Epidemiol Biomarkers Prev. 2015;24:1190–8. 25994738 10.1158/1055-9965.EPI-14-1389 PMC4526388

[15] Fechete O Ungureanu L Șenilă S. Risk factors for melanoma and skin health behaviour: an analysis on Romanian melanoma patients. Oncol Lett. 2019;17:4139–44. 30944607 10.3892/ol.2018.9737 PMC6444336

[16] Kyprianou D Charalambidou I Famojuro O Wang H Su D Farazi PA. Knowledge and attitudes of cypriots on melanoma prevention: is there a public health concern? BMC Public Health. 2022;22:53. 34998365 10.1186/s12889-021-12324-0 PMC8742933

[17] Coups EJ Geller AC Weinstock MA Heckman CJ Manne SL. Prevalence and correlates of skin cancer screening among middle-aged and older white adults in the United States. Am J Med. 2010;123:439–45. 20399321 10.1016/j.amjmed.2009.10.014 PMC2858071

[18] Kasparian NA Bränström R Chang YM. Skin examination behavior: the role of melanoma history, skin type, psychosocial factors, and region of residence in determining clinical and self-conducted skin examination. Arch Dermatol. 2012;148:1142–51. 22801744 10.1001/archdermatol.2012.1817 PMC4965805

[19] Saitta P Cohen DE Rigel D Grekin SK Brancaccio R. The frequency of self-skin examination and full body skin examination in dermatologists. J Clin Aesthet Dermatol. 2011;4:20–4. PMC3140903 21779412

[20] Puig S Malvehy J. Monitoring patients with multiple nevi. Dermatol Clin. 2013;31:565–77. 24075545 10.1016/j.det.2013.06.004

[21] Kaminska-Winciorek G Wydmanski J Gajda M Tukiendorf A. Melanoma awareness and prevalence of dermoscopic examination among internet users: a cross-sectional survey. Postepy Dermatol Alergol. 2016;33:421–8. 28035218 10.5114/pdia.2016.63297 PMC5183778

[22] Kasparian NA McLoone JK Meiser B Butow PN Simpson JM Mann GJ. Skin cancer screening behaviours among individuals with a strong family history of malignant melanoma. Br J Cancer. 2010;103:1502–9. 20978504 10.1038/sj.bjc.6605942 PMC2990585

[23] Demidov L Samoylenko I Vand N Utyashev I Shubina I Sinelnikov I. Screening for melanoma and other skin cancer shows a higher early melanoma incidence: social educational program “Life Fear-Free.”. Dermatopathology (Basel). 2021;8:54–68. 33803969 10.3390/dermatopathology8010011 PMC8008317

[24] Seetan K Khamees A Migdadi A. Knowledge, attitude, and practice toward skin cancer prevention and detection among jordanian medical students: a cross-sectional study. J Skin Cancer. 2022;2022:6989827. 35198247 10.1155/2022/6989827 PMC8860529

[25] Karasiewicz M Chawłowska E Lipiak A Więckowska B. A step towards understanding and tackling health inequalities: the use of secondary prevention services and the need for health promotion in a rural setting. Int J Environ Res Public Health. 2021;18:8492. 34444237 10.3390/ijerph18168492 PMC8394776

[26] Uğrlu Z Işik SA Balanuye B Budak E Elbaş NO Kav S. Awareness of skin cancer, prevention, and early detection among Turkish University Students. Asia Pac J Oncol Nurs. 2016;3:93–7. 27981144 10.4103/2347-5625.170969 PMC5123539

[27] Bergenmar M Törnberg S Brandberg Y. Factors related to non-attendance in a population based melanoma screening program. Psychooncology. 1997;6:218–26. 9313288 10.1002/(SICI)1099-1611(199709)6:3<218::AID-PON265>3.0.CO;2-G

[28] Reyes-Marcelino G Tabbakh T Espinoza D. Prevalence of skin examination behaviours among Australians over time. Cancer Epidemiol. 2021;70:101874. 33341599 10.1016/j.canep.2020.101874

[29] Coups EJ Xu B Heckman CJ Manne SL Stapleton JL. Physician skin cancer screening among U.S.military veterans: Results from the National Health Interview Survey. PLoS One. 2021;16:e0251785. 34003851 10.1371/journal.pone.0251785 PMC8130944

[30] Renzi C Mastroeni S Mannooranparampil TJ Passarelli F Caggiati A Pasquini P. Skin cancer knowledge and preventive behaviors among patients with a recent history of cutaneous squamous cell carcinoma. Dermatology. 2008;217:74–80. 18424897 10.1159/000127389

